# Rebuilding the Mucociliary Apparatus in ECRS: TSLP/IL-33 Signaling Synergy and the Residual Molecular Scar of *DNASE1L3* Following IL-4/13 Blockade

**DOI:** 10.3390/cells15100911

**Published:** 2026-05-15

**Authors:** Rikuto Fujita, Takashi Ishino, Takashi Oda, Tomohiro Kawasumi, Manabu Nishida, Yuichiro Horibe, Nobuyuki Chikuie, Takayuki Taruya, Takao Hamamoto, Tsutomu Ueda, Sachio Takeno

**Affiliations:** Department of Otorhinolaryngology, Head and Neck Surgery, Graduate School of Biomedical Sciences, Hiroshima University, Kasumi 1-2-3, Minami-ku, Hiroshima 734-8551, Japan; rikto@hiroshima-u.ac.jp (R.F.); odataka@hiroshima-u.ac.jp (T.O.); kwtm2022@hiroshima-u.ac.jp (T.K.); nm1027@hiroshima-u.ac.jp (M.N.); horibey@hiroshima-u.ac.jp (Y.H.); housejak@hiroshima-u.ac.jp (N.C.); ttaruya@hiroshima-u.ac.jp (T.T.); takao0320@hiroshima-u.ac.jp (T.H.); uedatsu@hiroshima-u.ac.jp (T.U.); takeno@hiroshima-u.ac.jp (S.T.)

**Keywords:** multiciliogenesis, thymic stromal lymphopoietin (TSLP), IL-33, type 2 inflammation, eosinophilic rhinosinusitis

## Abstract

Background: Eosinophilic chronic rhinosinusitis (ECRS) is characterized by refractory nasal polyps and severely impaired mucociliary clearance (MCC). The molecular mechanisms underlying the modulation of mucociliogenesis following IL-4/13 blockade with dupilumab remain poorly understood, notwithstanding its proven clinical efficacy. Methods: Bulk RNA Barcoding and sequencing (BRB-seq) was performed on nasal polyp tissues collected from healthy controls (*n* = 6), patients with non-ECRS (*n* = 8), and patients with ECRS both before and four weeks after dupilumab treatment (*n* = 9) to identify the early molecular drivers underlying ciliary regeneration. Comprehensive gene-set scoring systems were developed to evaluate multiciliogenesis master regulators, master regulators of core/ciliary planar cell polarity (PCP) and PCP components. Interaction scores for epithelial-derived cytokines—thymic stromal lymphopoietin (TSLP), IL-25, and IL-33—were calculated based on ligand and cognate receptor subunit expression. Results: The ciliary master regulatory hierarchy (e.g., *FOXJ1*, *RFX2/3*), PCP components (*CELSR1* and the ciliogenesis and planar polarity effector (CPLANE) module: *FUZ*, *INTU*, *WDPCP*), and structural ciliogenesis pathways were robustly restored following IL-4/13 blockade. The TSLP interaction score correlated with global mucosal damage, serving as a trigger for compensatory multiciliogenesis. The pre-treatment IL-33 interaction score emerged as a significant predictor of transcriptomic ciliary recovery (*p* < 0.05). *DNASE1L3*—the primary endonuclease for degrading eosinophilic extracellular traps (EETs)—remained persistently downregulated post-treatment. Conclusions: IL-4/13 blockade successfully restores the structural and directional “hardware” of the respiratory epithelium but fails to rectify the enzymatic “software” required for mucus degradation. This “residual molecular scar” may explain the persistent mucus hyperviscosity observed in some ECRS patients even after clinical polyp resolution.

## 1. Introduction

Mucociliary clearance (MCC) within the upper and lower respiratory tracts constitutes a vital primary defense, safeguarding the mucosal tissue against a diverse array of environmental exposures, including smoke, bio-aerosols, dust, fungi, and toxic pollutants. This homeostatic mechanism is sustained through the orchestrated synergy of autonomous ciliary beating, planar cell polarity (PCP)-mediated coordination, and the precise biochemical and rheological properties of the surface mucus [1,2].

At the ultrastructural level, motile cilia are characterized by a conserved 9+2 axonemal arrangement, where nine peripheral doublet microtubules encircle a central apparatus—a complex comprising a central microtubule pair and its associated projections [3,4,5]. The molecular architecture of the cilium is organized into several functionally distinct regions: the basal body, which anchors the cilium to the apical membrane via a modified centriole; the transition zone, a specialized gatekeeper at the ciliary base that regulates protein trafficking; and the axoneme, the core skeleton. This framework is equipped with essential motor and regulatory complexes, including outer and inner dynein arms (ODAs/IDAs), radial spokes, and the nexin-dynein regulatory complex (N-DRC) [5,6].

The genesis of multiciliated cells (MCCs) proceeds through an intricately regulated, multi-step developmental program encompassing centriole amplification, apical migration, docking, and axonemal assembly. This lineage is governed by a stringent gene-regulatory hierarchy initiated by tumor protein P73 (TP73) under the suppression of NOTCH signaling. The progression of multiciliogenesis is further characterized by the concerted action of a sophisticated and highly coordinated regulatory landscape. This intricate program involves an essential repertoire of transcription factors and regulatory elements—including TP73, multiciliate differentiation and DNA synthesis associated (MCIDAS), E2F transcription factor 4/5 (E2F4/5), development of ependymal and urothelial cilia 1 (DEUP1), cell division cycle 20B (CDC20B), MYB proto-oncogene (MYB), cyclin O (CCNO), forkhead box J1 (FOXJ1), forkhead box N4 (FOXN4), and the regulatory factor X (RFX) family—which collectively orchestrate the complex transition from progenitor commitment to the functional maturation of motile cilia [5,7,8,9,10,11,12,13,14,15].

The program culminates in the activation of FOXJ1 and the RFX family, which together govern axonemal assembly and the functional maturation [5,7,8,9,10,11,12,13,14,15]. Specifically, FOXJ1 regulates genes critical for axoneme assembly, dynein arm components, radial spoke proteins, and intraflagellar transport (IFT) [5,16]. Similarly, RFX2 and RFX3 redundantly control multiciliogenesis by regulating a wide array of target genes, including those encoding IFT components (both IFT-A and IFT-B complexes), axonemal structures, the BBSome complex, and centrosomal proteins [5,16,17,18]. These components are indispensable for the sustained motility and homeostatic maintenance of motile cilia.

PCP represents a pivotal determinant for the coordinated motility of MCCs within the airway epithelium. The PCP signaling pathway governs the proximal–distal orientation of cilia within individual MCCs, a process characterized by the asymmetric distribution of core PCP proteins across the apical surface [19]. This precise spatial localization facilitates unified, vectorial ciliary beating, which is an absolute prerequisite for efficient MCC [1,19].

The molecular framework of PCP is established by genes encoding integral membrane proteins—such as Frizzled (FZD), Vang-like (VANGL), and Cadherin EGF LAG seven-pass G-type receptor (CELSR)—which act in concert with peripheral membrane proteins, including dishevelled (DVL), prickle planar cell polarity protein (PRICKLE), and ankyrin repeat domain 6 (ANKRD6; Diego). These core components further recruit planar polarity effectors (PPE) factors, such as inturned (INTU), fuzzy (FUZ), and WD repeat containing planar cell polarity effector (WDPCP; Fritz), to define the architectural polarity of the cell [19,20,21].

The Frizzled/Starry night (Fz/Stan) axis, a hierarchical regulator of PCP across various tissues, has been significantly implicated in the impairment of mucociliary transport when dysregulated. Collectively, these PCP-driven mechanisms interface with multiciliogenesis programs to forge a highly ordered epithelial landscape, ensuring that hundreds of cilia per cell oscillate synchronously and with correct directional bias. Consequently, perturbations in PCP signaling—whether of genetic or acquired etiology—undermine this organizational integrity, contributing to the pathogenesis of respiratory conditions defined by defective MCC.

In the context of MCC, the gel-forming mucins, MUC5AC and MUC5B, serve as fundamental biological determinants of efficient airway transport by modulating the viscoelasticity and rheological properties of the surface mucus [22,23,24].

Chronic inflammatory pathologies of the upper and lower airways, such as eosinophilic chronic rhinosinusitis (ECRS) and bronchial asthma, are predominantly characterized by a Type 2 inflammatory milieu. This environment is defined by the overproduction of IL-4, IL-5, and IL-13, coupled with robust eosinophilic infiltration into local tissues and peripheral blood eosinophilia. These conditions exhibit a marked decline in MCC, resulting from the dual impact of impaired motile ciliogenesis and pathological alterations in mucus composition [2,25,26]. Specifically, IL-13 is known to antagonize *FOXJ1* expression via the signal transducer and activator of transcription 6 (STAT6) signaling axis and downregulate RFX3, which ultimately precipitates a significant loss of functional ciliated cells [27,28]. Furthermore, attenuated expression of PCP-associated genes and proteins has been documented in nasal polyps, leading to the disruption of PCP and a consequent reduction in mucociliary transport velocity [2,26].

Pathological mucus production and altered compositional stoichiometry are likewise inextricably linked to Type 2 inflammation. IL-13 facilitates the expression of arachidonate 15-lipoxygenase (*ALOX15*) and the synthesis of its lipid metabolite, 15-hydroxyeicosatetraenoic acid-phosphatidylethanolamine (15-HETE-PE), thereby augmenting *MUC5AC* expression [29,30]. Simultaneously, IL-13 promotes SAM pointed domain containing ETS transcription factor (SPDEF) expression via STAT6 to drive goblet cell metaplasia (GCM)—a requisite process for excessive mucus secretion [31,32]. Periostin (encoded by *POSTN*) also contributes to the upregulation of MUC5AC [33]. The resulting imbalance—characterized by elevated MUC5AC and diminished MUC5B in asthma [28,34], or the elevated levels of both mucins in chronic rhinosinusitis (CRS)—leads to profound mucus hypersecretion and the catastrophic failure of the mucociliary apparatus [35,36].

Beyond mucin stoichiometry, eosinophilic mucin is characterized by extensive fibrin polymerization [37] and the dissemination of extracellular DNA networks via ETosis. The resulting eosinophilic extracellular traps (EETs), composed of histone H1-associated DNA networks, form a structural scaffold that profoundly increases mucus hyperviscosity [38,39,40]. The molecular regulation of this fibrinostasis is closely linked to the expression of serpin family E member 1 (*SERPINE1*; encoding plasminogen activator inhibitor-1, PAI-1) and plasminogen activator, tissue type (*PLAT*; encoding tissue-type plasminogen activator, t-PA). In chronic rhinosinusitis with nasal polyps (CRSwNP), the upregulation of *SERPINE1* and the concomitant downregulation of PLAT precipitate excessive fibrin polymerization, further compounding the tenacity of the eosinophilic mucus [41]. Furthermore, these DNA traps, which are primarily composed of extracellular microparticle-associated chromatin, are typically targeted for digestion by the endonuclease deoxyribonuclease 1 like 3 (DNASE1L3) [42,43]. Consequently, a deficit in this degradative pathway may play a pivotal role in the persistent hyperviscosity observed in ECRS.

The convergence of these pathological mechanisms—impaired ciliary function, disrupted PCP, and altered mucus rheology—synergistically reduces mucociliary transport efficiency in patients with CRS compared to healthy individuals [44].

Dupilumab, a recombinant human immunoglobulin G4 (IgG4) monoclonal antibody targeting the IL-4Rα, inhibits both IL-4 and IL-13 signaling by binding to the shared IL-4Rα component of their respective receptor complexes. Clinically, it has demonstrated transformative efficacy in reducing polyp burden and alleviating the core pathophysiology of CRSwNP [45,46,47]. However, while the association between Type 2 inflammation and ciliary dysfunction is increasingly recognized, the impact of pharmacological IL-4/13 blockade on the comprehensive genomic landscape of MCC—specifically the recovery of ciliary programs and the modulation of mucus rheology—remains to be fully elucidated.

This study aims to evaluate the early transcriptomic modulation of genes governing motile cilia formation, PCP, and the properties of high-viscosity eosinophilic mucin following the suppression of Type 2 inflammation with dupilumab, thereby elucidating the early molecular mechanisms underlying mucociliary dysfunction in CRS.

## 2. Materials and Methods

### 2.1. Patient Recruitment and Specimen Collection

#### 2.1.1. Study Population and Ethical Oversight

This prospective study enrolled patients diagnosed with CRSwNP at Hiroshima University Hospital between June 2021 and October 2023. The study cohort comprised individuals with non-ECRS (*n* = 8) and those with ECRS (*n* = 9). Control specimens (Ctrl, *n* = 6) were sourced from patients undergoing surgery for anatomical abnormalities (e.g., septal deviation) who exhibited no evidence of inflammatory mucosal changes or concurrent bacterial infections. Clinical characteristics of the study population are summarized in Table 1.

#### 2.1.2. Diagnostic Criteria for ECRS

The diagnosis of ECRS was stringently established in accordance with the Japanese Epidemiological Survey of Refractory Eosinophilic Chronic Rhinosinusitis (JESREC) criteria, requiring a total clinical score of ≥11 and a tissue eosinophil count of ≥70 cells per high-power field within the nasal polyps [45]. The clinical score was determined by a composite assessment of the following parameters:

- Disease Distribution: Bilateral involvement (3 points).
- Presence of Nasal Polyps: (2 points).
- Radiological Findings: Computed tomography (CT) showing ethmoid-dominant opacification (ethmoid/maxillary ratio ≥ 1; 2 points).

Peripheral Eosinophilia:

- 2% < eosinophils ≤ 5% (4 points);
- 5% < eosinophils ≤ 10% (8 points);
- eosinophils>10% (10 points).

#### 2.1.3. Therapeutic Protocol and Specimen Acquisition

Refractory ECRS patients (polyp score ≥ 5; history of ESS) received biweekly dupilumab. Nasal polyp tissues were collected at baseline (PreECRS) and four weeks post-initiation (PostECRS). This 4-week interval was strategically selected to facilitate longitudinal comparison within the same mucosal niche before rapid clinical resolution precluded site-specific topographical analysis. This paired sampling (*n* = 9) minimized inter-individual variability and captured the early molecular shifts in mucosal remodeling.

### 2.2. Library Preparation via Modified Bulk RNA Barcoding and Sequencing (BRB-Seq)

Transcriptomic profiling was performed using modified BRB-seq [48]. First-strand cDNA was synthesized with a barcoded oligo-dT primer (5′-GCCGGTAATACGACTCACTATAGGGAGTTCTACAGTCCGACGATCNNNNNNNNNNCCCCCCCCCTTTTTTTTTTTTTTTTTTTTTTTTV-3′) containing a 10 bp UMI and 9 bp cell barcode. After second-strand synthesis (NEB, Ipswich, MA, USA, #E6111) and tagmentation via an in-house MEDS-B Tn5 transposase [49,50], libraries were amplified for 10 PCR cycles using Phusion High-Fidelity DNA Polymerase (Thermo Scientific, Waltham, MA, USA, #M0530): The following primers were employed for the amplification process:

- Forward: 5′-AATGATACGGCGACCACCGAGATCTACACindexGTTCAGAGTTCTACAGTCCGA-3′
- Reverse: 5′-CAAGCAGAAGACGGCATACGAGATindexGTCTCGTGGGCTCGGAGATGT-3′

Sequencing was executed on the Illumina NovaSeq 6000 platform (Illumina, Inc., San Diego, CA, USA), generating a 15 bp barcode read (Read 1) and an 81 bp insert read (Read 2).

### 2.3. Bioinformatic Processing of BRB-Seq Data

Bioinformatics analysis of the BRB-seq libraries was performed through a structured computational pipeline. Initially, the barcode read (Read 1) was extracted using UMI-tools (ver. 1.1.1) with the following command: umi_tools extract -I read1.fastq --read2-in=read2.fastq --bc-pattern=NNNNNNNNNNCCCCCCCCC --read2-stdout. Subsequently, adapter sequences and low-quality bases were excised using Trim Galore (ver. 0.6.7), with a minimum read length threshold of 20 bp.

The processed reads were aligned to the GRCh38 human reference genome utilizing HISAT2 (ver. 2.2.1). Gene-level quantification was performed via featureCounts (ver. 2.0.1), and PCR duplicates were collapsed based on Unique Molecular Identifiers (UMIs) using the unique method in UMI-tools (--per-gene --per-cell --gene-tag=XT). Finally, read count normalization was conducted using DESeq2 (ver. 1.34.0) to facilitate downstream comparative analyses.

### 2.4. Curation of Investigated Gene Sets

To evaluate the epithelial landscape of ECRS, we curated comprehensive gene modules subcategorized by functional roles:

- Multiciliogenesis Master Regulators (*n* = 12; Master genes): Including TP73, MCIDAS, E2F4/5, MYB, CCNO, DEUP1, CDC20B, FOXJ1, FOXN4, and RFX2/3.
- PCP Components (*n* = 17; PCP genes): Core and ciliary effector genes.
- Ciliogenesis Regulators (*n* = 143; Ciliogenesis genes): Broad structural and functional factors.
- Mucus Pathophysiology & GCM (*n* = 25; Mucus-associated genes): Including MUC5AC, MUC5B, and markers of hyper-secretion and viscosity.
- Epithelial Cytokines & Receptors: Including thymic stromal lymphopoietin (TSLP), IL25, IL33, and their respective cognate receptor subunits.

These gene sets used for ciliary, epithelial, and inflammatory modules were derived from our in-house curated annotations based on previously published literature. Full gene lists are provided in Supplementary Table S1 to ensure reproducibility.

### 2.5. Statistical Analysis, Framework and Scoring Systems

To compare ciliary and PCP scores among the four study groups (Ctrl, nonECRS, PreECRS, and PostECRS), linear mixed-effects models (LMM) were employed using the lmerTest and lme4 packages. In these models, ‘Group’ was treated as a fixed effect, while ‘PatientID’ was incorporated as a random effect to account for both inter-individual variability and the paired nature of the PreECRS and PostECRS samples. For post hoc analysis, Tukey’s Honest Significant Difference (HSD) test was performed using the emmeans package to adjust for multiple pairwise comparisons. The association between epithelial cytokine interaction scores and ciliary/PCP metrics was assessed using marginal *R* (*Rm*) based on Nakagawa’s coefficient of determination (*R*^2^). The statistical significance of these associations was determined via LMM to further account for inter-individual variability.

Differential Expression Analysis was conducted using the DESeq2 package. Differentially expressed genes (DEGs) were identified using a significance threshold of a *p_adjusted_* < 0.05 and an absolute log_2_ fold change > 1.

Cellular Program Scoring: To quantitatively assess specific cellular programs, we calculated Cilia Master, Core PCP, and Cilia PCP scores. These scores represent the arithmetic mean of the log_2_ transformed counts per million (log_2_(counts per million (CPM) + 1)) for the following gene sets:

- Cilia Master Score: TP73, MCIDAS, E2F4/5, CCNO, DEUP1, MYB, FOXJ1, FOXN4, RFX2/3.
- Core PCP Score: CELSR1–3, FZD3/6, VANGL1–2, DVL1–3, PRICKLE1–4, ANKRD6.
- Cilia PCP Score: FUZ, INTU, WDPCP.

Cytokine Interaction Scores: Interaction scores for epithelial-derived cytokines (IL-25, IL-33, and TSLP) were derived to estimate signaling potential. The score was defined as the product of the ligand’s expression and the mean expression of its corresponding receptor subunits (e.g., $TSLP\times \frac{CRLF2+IL7R}{2}$).

In all analyses, *p* < 0.05 was considered statistically significant, and all statistical computations were executed in the R environment (version 4.3.3), utilizing the DESeq2 (ver. 1.42.1), lme4 (ver. 1.1-35.5), lmerTest (ver. 3.1-3), emmeans (ver. 2.0.3), ggplot2 (ver. 3.5.2), and ggpubr (ver. 0.6.1) packages.

## 3. Results

### 3.1. Expression Profiles of Multiciliogenesis Master Regulators

**Figure 1:**
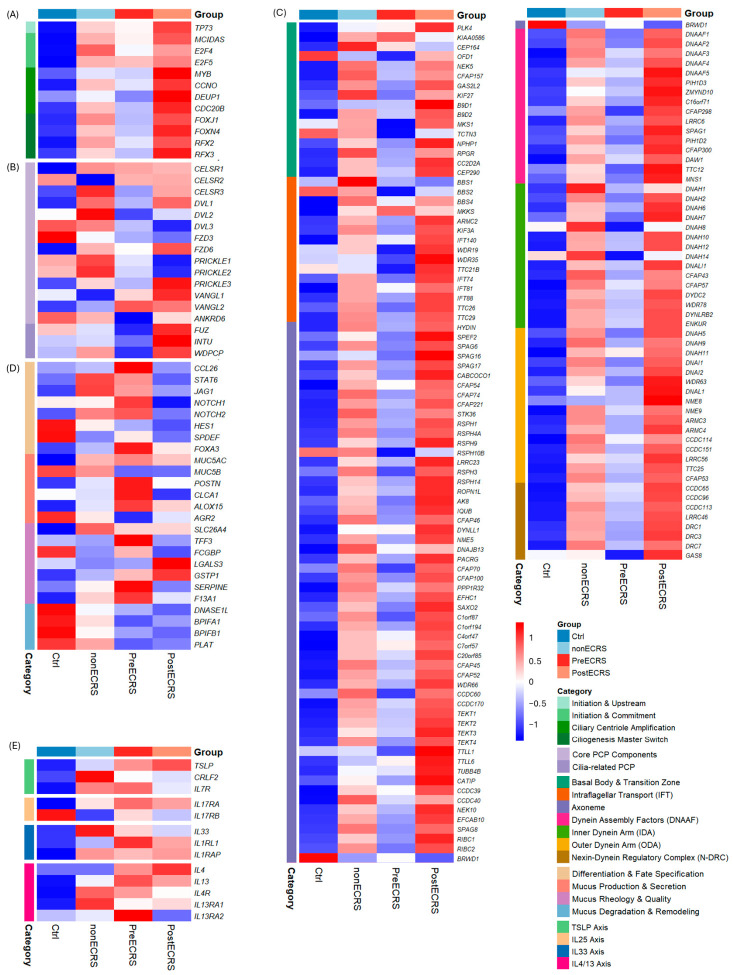
Transcriptional Profiles of Ciliogenesis, PCP, Mucus, and Type 2 Inflammation in CRSwNP. Heatmaps displaying relative gene expression (*Z*-score) of specific gene sets across control (Ctrl), non-eosinophilic chronic rhinosinusitis (nonECRS), and eosinophilic chronic rhinosinusitis with nasal polyps (PreECRS and PostECRS) groups. Rows are categorized by biological function or structural component. The color scale indicates standard deviations above (red) and below (blue) the mean. (**A**) Master genes: Regulators of multiciliogenesis staged by initiation, commitment, centriole amplification, and maturation. (**B**) PCP genes: Core and cilia-related components governing planar cell polarity. (**C**) Ciliogenesis genes: Structural and regulatory components including the basal body, IFT, axoneme, dynein arms (IDA/ODA), and N-DRC. (**D**) Mucus-associated genes: Factors involved in goblet cell differentiation, mucus secretion, rheology, and degradation/remodeling. (**E**) Epithelial-derived & IL-4/13 Cytokines/Receptors: Key signaling components of the TSLP, IL-25, IL-33, and IL-4/13 axes.

The transcriptional landscape of Master genes governing multiciliated cell formation exhibited marked induction in both the non-ECRS and PostECRS cohorts, whereas the PreECRS group displayed consistently lower expression levels relative to both counterparts (Figure 1A). Notably, these regulatory factors reached their nadir in the control (Ctrl) group and were robustly upregulated in the PostECRS group. Cross-group comparisons identified significant DEGs within the PostECRS vs. Ctrl axis, including *TP73*, *MYB*, *CCNO*, *CDC20B*, *FOXJ1*, *RFX2*, and *RFX3* (Table 2).

### 3.2. Divergent Modulation of PCP Components

Regarding the architectural components of PCP, a substantial upregulation of *DVL1*, *FZD6*, *PRICKLE3*, *VANGL1/2*, *FUZ*, *INTU*, and *WDPCP* was observed in the PostECRS group; conversely, these genes—except for *VANGL1/2*—were significantly suppressed in the PreECRS state (Figure 1B). In the control group, *DVL3* and *FZD3* exhibited preferential upregulation compared to other pathological states. Interestingly, *DVL1* and *DVL3* displayed distinct behavioral trajectories between the Ctrl and PostECRS groups, a pattern similarly observed for the *PRICKLE1/2* and *PRICKLE3* isoforms. In the comparative cross-analysis, *CELSR1* emerged as the exclusive DEG, identified only within the PreECRS vs. Ctrl comparison (Supplementary Table S2A).

### 3.3. Comprehensive Activation of Mucociliogenesis Pathways

A congruent trend was observed in the genes associated with mucociliogenesis, paralleling the expression patterns of the master regulators (Figure 1C). All functional categories within this module were profoundly upregulated in the PostECRS group relative to all other cohorts. Quantitative analysis of DEGs further underscored this shift: the “PostECRS vs. Ctrl” comparison yielded 81 DEGs, a figure that sharply contrasted with the 12 and 9 DEGs identified in the “non-ECRS vs. Ctrl” and “PreECRS vs. Ctrl” comparisons, respectively (Supplementary Table S2B).

### 3.4. Goblet Cell Metaplasia and Mucus Pathophysiology

Evaluation of the gene modules associated with GCM and mucus hyperviscosity revealed that *CCL26*—a pivotal type 2 inflammatory inducer responsible for eosinophil recruitment and the subsequent activation of *STAT6*, *JAG1*, and *NOTCH1/2*—was strongly elevated in the PreECRS group (Figure 1D). In contrast, the “Mucus Degradation & Remodeling” category exhibited sustained downregulation in both the PreECRS and PostECRS groups compared to controls. While the DEGs for “PreECRS vs. Ctrl” and “PostECRS vs. Ctrl” showed substantial overlap, distinct expression profiles were maintained for *CCL26*, *MUC5AC*, *SLC26A4*, *GSTP1*, and *SERPINE1* (Table 2B).

### 3.5. Epithelial Cytokine and Receptor Profiling

Transcriptomic profiling of epithelial-derived cytokines demonstrated that the PreECRS group upregulated most of these genes relative to controls, with the notable exception of *IL25*, which remained undetected across all cohorts (Figure 1E). Following dupilumab treatment (PostECRS), the expression of *IL4R*, *IL13RA1*, and the receptors for TSLP and IL-25 decreased compared to the PreECRS state. *IL1RL1* was identified as the sole common DEG across the PreECRS vs. Ctrl, PostECRS vs. Ctrl, and PreECRS vs. non-ECRS comparisons (Supplementary Table S2C).

### 3.6. Quantitative Assessment of Ciliary and PCP Scores

**Figure 2:**
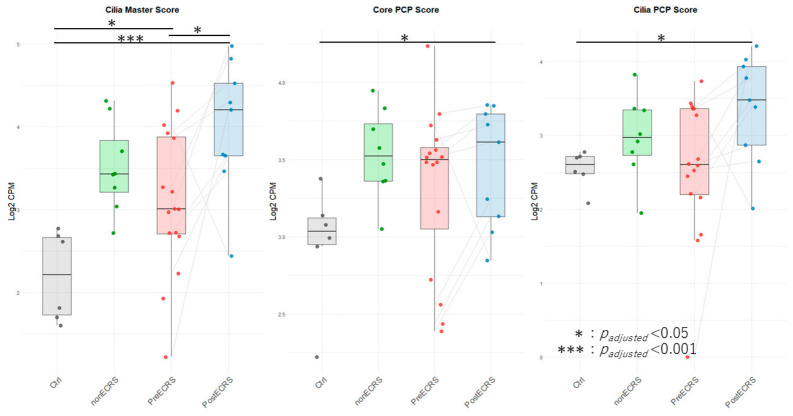
Quantitative Analysis of Ciliary and PCP Gene Expression Scores. Aggregate log_2_ CPM scores for Cilia Master, core PCP, and cilia PCP across study groups. Box plots show median/IQR; lines connect paired Pre- and Post-dupilumab samples. The Cilia Master score (*p*_adjusted_ = 0, unpaired), core PCP score (*p*_adjusted_ = 0.035, unpaired), and cilia PCP score (*p*_adjusted_ = 0.01, unpaired) all exhibited significant elevation in the PostECRS vs. Ctrl comparison (Figure 2). Furthermore, significant increases in the Cilia Master score were observed in both the PreECRS vs. PostECRS (*p*_adjusted_ = 0.042, paired) and PreECRS vs. Ctrl (*p*_adjusted_ = 0.012, unpaired) comparisons.

The Cilia Master score (*p* = 0, unpaired), core PCP score (*p* = 0.035, unpaired), and cilia PCP score (*p* = 0.01, unpaired) all exhibited significant elevation in the PostECRS vs. Ctrl comparison (Figure 2). Furthermore, significant increases in the Cilia Master score were observed in both the PreECRS vs. PostECRS (*p* = 0.042, paired) and PreECRS vs. Ctrl (*p* = 0.012, unpaired) comparisons.

### 3.7. Correlation Analysis of TSLP and IL-33 Interaction Scores

**Figure 3:**
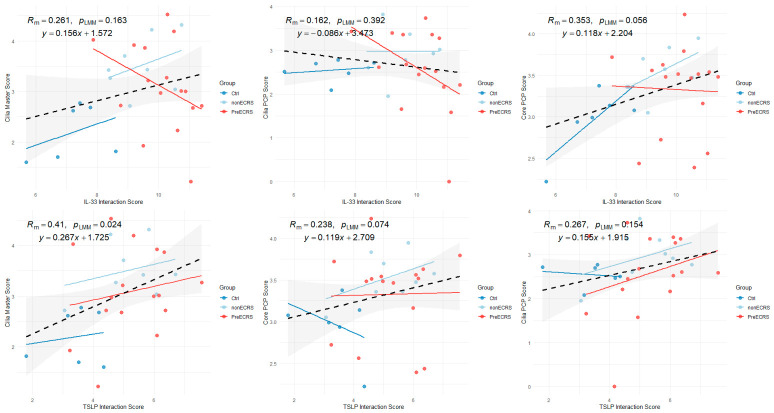
Correlations between Epithelial Cytokine Scores and Ciliary/PCP Metrics. Scatter plots showing relationships between cytokine interaction scores (IL-33, TSLP) and ciliary/PCP metrics across Ctrl, nonECRS, and PreECRS groups. Total Correlation: Dashed black lines indicate regression across all samples; linear mixed-effects model, *p_LMM_*-values, and equations are shown. Group Dynamics: Solid lines (Ctrl: blue; nonECRS: light blue; PreECRS: red) represent group-specific regressions with 95% confidence intervals. A significant correlation was observed between the TSLP interaction score and Cilia Master score (*p_LMM_* = 0.024), suggesting a link between TSLP signaling and multiciliogenesis regulation. *Rm* denotes the marginal correlation coefficient representing the fixed effects of the cytokines, and *P_LMM_* represents the significance level accounting for random effects (PatientID). The grey shaded area represents the 95% confidence interval.

In assessing the relationship between epithelial cytokine interaction scores and ciliary markers, the TSLP interaction score demonstrated a significantly moderate positive correlation with the Cilia Master score within the context of overall disease activity (*p* < 0.05) (Figure 3).

**Figure 4:**
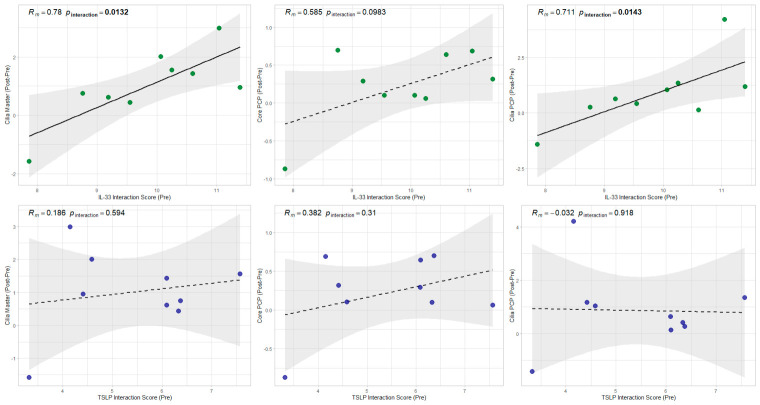
Correlation between Epithelial Cytokine Scores and the recovery of Ciliary/PCP Metrics during dupilumab administration. Scatter plots illustrate the association between baseline cytokine interaction scores (PreECRS) (x-axes) and ciliary functional scores (y-axes). Significant correlations (*p_interaction_* < 0.05) are indicated by solid regression lines and bold *p* values, while non-significant trends (*p_nteraction_* ≥ 0.05) are shown with dashed lines. The shaded regions represent the 95% confidence intervals of the linear regression. *Rm* denotes the marginal correlation coefficient and significance levels (*p_interaction_*) were calculated using linear mixed-effects model. The grey shaded area represents the 95% confidence interval.

To evaluate the prognostic value of mucosal cytokines in the dupilumab administration, we employed linear mixed-effects models (LMM) to analyze the impact of baseline levels and longitudinal changes in IL-33 and TSLP on the recovery of Ciliary Master and Core/Cilia PCP gene scores. These analyses revealed that the baseline IL-33 interaction score—specifically through its interaction with the treatment condition—significantly predicted the magnitude of recovery (Post-Pre) in both Ciliary Master and Ciliary PCP scores (*p_interaction_* < 0.05; Figure 4). Conversely, treatment-induced longitudinal fluctuations in IL-33 or TSLP showed no significant tracking with any of the ciliary and PCP parameters, suggesting that initial IL-33 levels, rather than real-time cytokine dynamics, dictate the mucosal regenerative potential (Supplementary Table S3). Furthermore, multivariate LMM analysis was performed to adjust for potential confounding clinical factors, including blood eosinophil count, total IgE, Lund-Mackay CT score, oral fractional exhaled nitric oxide (FeNO), tissue eosinophilia, and JESREC score (Supplementary Table S4). This confirmed that baseline IL-33 remained a significant and independent predictor for the recovery of cilia and PCP formation, demonstrating its superior prognostic value compared to these conventional clinical markers.

## 4. Discussion

CRS is categorized into CRSwNP and chronic rhinosinusitis without nasal polyps (CRSsNP), with the former further stratified into ECRS and non-ECRS phenotypes based on tissue eosinophilia [45,51]. ECRS, driven by Type 2 inflammation, is characterized by ethmoid-dominant opacification, viscous eosinophilic mucin, multifaceted polyp formation, and olfactory impairment, frequently manifesting in tandem with bronchial asthma.

Regarding the master regulatory genes essential for multiciliogenesis, the PostECRS group demonstrated a comprehensive upregulation of these factors, whereas the non-ECRS and PreECRS cohorts exhibited a more nuanced, moderate induction across select genes. Notably, a significant elevation of the ciliary master score was observed in both PreECRS and PostECRS relative to controls, as well as in the comparison between PostECRS and PreECRS. These findings imply that both non-ECRS and ECRS environments provide a milieu that, to varying degrees, favors the initiation of mucociliogenesis. While bulk RNA-seq captures the collective signal from the tissue, the coordinated upregulation of master regulators such as FOXJ1 and RFX suggests a robust activation of the multiciliogenesis program within the epithelial compartment. This transcriptional surge likely arises from the extensive attrition of MCCs on the sinus mucosa, precipitated by a myriad of stressors including bacterial infection, environmental pollutants, fungus, viruses, and the underlying type 2 inflammatory cascade.

In our correlation analysis, the TSLP Interaction Score identified a moderate positive association with ciliary master genes. This association potentially underscores a compensatory reparative mechanism—a vital attempt by the epithelium to regenerate its ciliary architecture in response to relentless inflammatory injury. Conversely, the profound downregulation of these genes in the control group reflects a state of mucosal quiescence, where the absence of significant damage obviates the need for such regenerative signaling.

A comparative analysis between PreECRS and PostECRS, specifically regarding the efficacy of IL-4/13 inhibition, clearly elucidated the modulation of master gene expression, thereby underscoring the potent promotion of mucociliogenesis upon the blockade of type 2 inflammation. This suggests a model in which controls remain quiescent and the inflamed mucosa initiates a compensatory repair demand; ultimately, IL-4/13 blockade enables the successful completion of this regenerative program. Under this framing, the peak expression observed post-treatment represents functional repair rather than pathological over-activation. Additionally, differentially expressed genes (DEGs) were exclusively identified in the PostECRS vs. Ctrl comparison, whereas no such significant deviation was observed between PreECRS and Ctrl. Given that administration of dupilumab shifts the pathological state of the sinus mucosa toward a physiological, “normal-like” phenotype in clinical settings, these findings provide a robust framework for understanding the regenerative mechanism of mucociliogenesis facilitated by dupilumab.

Intriguingly, while TSLP serves as a reflection of global disease activity, an evaluation of epithelial-derived cytokines in treatment-naïve ECRS (PreECRS) and their longitudinal shifts post-treatment revealed that the IL-33 interaction score in PreECRS was significantly correlated with the objective recovery of Cilia Master scores. While IL-33 activates group 2 innate lymphoid cells (ILC2s), TSLP acts synergistically to augment the proliferation and type 2 cytokine secretion of these IL-33-stimulated ILC2s [52,53]. Notably, the concurrent stimulation with TSLP and IL-33 elicits an approximate ten-fold increase in IL-5/13 production by ILC2s compared with IL-33 alone, whereas TSLP itself does not independently induce significant IL-5/13 production [53]. As IL-13 inhibits FOXJ1 expression via the STAT6 signaling pathway, leading to impaired motile cilia formation [27], the administration of dupilumab restores cilia master gene expression by neutralizing this type 2 inflammatory axis. Consequently, while elevated TSLP signaling indicates the magnitude of type 2 inflammation and the attendant need for mucociliary repair, the IL-33 signal emerges as a pivotal and more direct predictor of functional ciliary recovery, owing to its indispensable synergistic role with TSLP in driving the ILC2-mediated production of IL-4/13.

Our study demonstrated that dupilumab treatment significantly upregulated an array of master gene expressions essential for motile cilia formation. In our analysis, FOXJ1 expression showed a robust upregulation in coordination with RFX2 and RFX3, further supporting their synergistic role in ciliary development. RFX2 and RFX3 act cooperatively or hierarchically with FOXJ1, which in turn has been shown to induce the expression of RFX2 and RFX3 during motile ciliogenesis [16,54,55,56]. While certain cilia-related genes are regulated solely by RFX2 [54], RFX3 alone does not induce the expression of ciliary genes and is thought to function as a cofactor for FOXJ1 [18,55]. Since IL-13 inhibits FOXJ1 expression via STAT6 signaling and simultaneously downregulates RFX3 expression [27,28], these combined effects, incorporating the modulation of RFX2, culminate in the suppression of motile ciliogenesis in CRSwNP, particularly within the ECRS endotype. Therefore, the effects on blockage of IL-13 can be interpreted as that controls remain quiescent, inflamed mucosa initiates a compensatory repair demand, and it enables the successful completion of the program. Under this framing, the peak expression observed post-treatment represents functional repair rather than pathological over-activation.

Planar cell polarity (PCP) is indispensable for the orchestrated movement of motile cilia; nonetheless, the mislocalization of PCP proteins has been documented in conditions such as cystic fibrosis, idiopathic pulmonary fibrosis, and CRS [1]. Our investigation revealed significant differential expressions among PCP-associated genes, most notably *CELSR1* and intriguing behavioral trajectories among PCP isoforms, particularly within the *DVL* and *PRICKLE* families. While *DVL3* was prominent in the control group, dupilumab treatment specifically augmented *DVL1*, echoing patterns observed in neural and sensory differentiation where specific isoforms govern distinct stages of maturation. This suggests that the post-treatment mucosa does not merely revert to a “control” state but rather recapitulates a developmental-like program of architectural remodeling. The preferential recovery of *CELSR1* and the ciliogenesis and planar polarity effector (CPLANE) module (*FUZ*, *INTU*, *WDPCP*) further underscores that IL-4/13 blockade restores the foundational molecular asymmetry required for directional ciliary transport. To address whether these transcriptional changes occur specifically within the airway epithelium, our previous work utilized scanning electron microscopy and protein-level validation [26]. We demonstrated that in NP mucosae, the physical presence of cilia is severely diminished and the protein distribution of WDPCP and FUZ is significantly reduced specifically within the cytoplasmic region of the remaining ciliated cells [26]. These findings provide epithelial-specific validation that the transcriptional downregulation observed in PreECRS corresponds to a loss of both structural integrity and key regulatory proteins within the epithelial layer itself.

Furthermore, a broad spectrum of PCP-associated genes exhibited significant downregulation in PreECRS compared to controls, with a subsequent robust upregulation following dupilumab treatment. Parallel to these findings, both the core PCP score and the ciliary PCP score were significantly elevated in PostECRS vs. Ctrl. Notably, the IL-33 interaction score in PreECRS exhibited a profound linkage to the eventual recovery of ciliary PCP scores.

These results imply that the mislocalization of PCP proteins in nasal polyps may stem from transcriptional perturbations precipitated by type 2 inflammation, and that the restoration of PCP architecture is significantly predicted by the baseline IL-33 interaction score in treatment-naïve ECRS (PreECRS). Additionally, we observed a diminished expression of FUZ, INTU, and WDPCP in PreECRS, which was successfully reversed after the administration of dupilumab. These genes, alongside FUZ, have been elucidated through recent proteomic and genetic analyses as integral components of the CPLANE gene module, which serves to recruit a specific subset of IFT-A proteins [57]. Furthermore, WDPCP is posited to serve as a scaffold at the ciliary transition zone to promote the assembly of multiprotein complexes essential for ciliogenesis [58]. Our earlier findings corroborated this at the protein level, showing that WDPCP protein distribution is markedly reduced in the cytoplasmic region of ciliated cells in CRSwNP patients [26]. Consequently, the expression patterns of WDPCP and INTU in this context likely pertain not only to PCP per se, but also to the broader process of ciliogenesis. While our transcriptomic data provides a robust molecular basis for PCP restoration, validating the asymmetric protein localization of these components remains an essential next step to confirm functional coordination, as the molecular asymmetry of PCP proteins is established antecedent to cilia formation [5]. In summary, our findings indicate that the expression of these PCP genes is adversely affected by type 2 inflammation, leading to a disruption of the physiological PCP landscape. The targeted blockade of IL-4/13 emerges as a pivotal determinant for the ideal reconstitution of PCP conformation, paralleling the recovery of ciliary master regulatory genes.

It is well-established that FOXJ1 and the RFX transcription factors serve as master regulators for an extensive repertoire of motile cilia-related genes, including the IFT machinery essential for ciliary assembly and homeostatic function [5,16,17,18]. Our findings demonstrated that genes associated with the IFT-A and IFT-B complexes, along with other critical structural categories—such as axonemal dyneins and radial spokes—were markedly upregulated following dupilumab administration. This suggests that IL-13 antagonism orchestrates a comprehensive restoration of ciliogenesis, encompassing not only the structural formation of cilia but also the logistical transport mechanisms and the motor apparatus required for ciliary motility.

Regarding the regenerative demand for multiciliated cell formation, the control group maintains mucosal homeostasis without the need for active cell synthesis. In contrast, the ECRS environment necessitates a robust regenerative response due to the chronic epithelial attrition precipitated by a myriad of external insults and type 2 inflammatory signaling. Since PreECRS displayed significantly lower expression levels of motile cilia-related genes compared to both the non-ECRS and PostECRS cohorts, these data corroborate the pathophysiological hallmark of severely impaired MCC in treatment-naïve ECRS. Furthermore, our results imply that non-ECRS may retain a higher degree of mucociliary functional integrity relative to the profoundly disrupted state observed in the ECRS phenotype. A critical consideration in interpreting our findings is the relationship between mRNA induction and functional recovery. We acknowledge that bulk RNA-seq cannot fully discriminate between increased per-cell transcription and an expansion of the ciliated cell population. While this study primarily evaluates the transcriptome, the robust and coordinated upregulation of the entire multiciliogenesis hierarchy—from master regulators (FOXJ1, RFXs) to structural axonemal components and IFT machinery—strongly suggests a comprehensive activation of the ciliary regenerative program. Furthermore, while it could be argued that bulk RNA-seq data might reflect shifts in cell populations (i.e., an increase in the number of ciliated cells) rather than purely intracellular transcriptional regulation, in the context of CRS, such a shift from inflammatory or secretory phenotypes toward a ciliated phenotype represents the fundamental mechanism of epithelial mucosal recovery. This interpretation is supported by our previous functional validation in CRSwNP patients, where the downregulation of these critical ciliogenesis genes—specifically IL-13-driven suppression of WDPCP—was directly responsible for the significant decrease in MCTV [26], confirming that the observed molecular deficits have direct physiological consequences. Although shorter than typical clinical evaluation periods, our focus on this early 4-week period allowed us to capture the “transcriptomic window”—the early molecular orchestration of ciliary regeneration that precedes definitive structural and functional recovery. Our findings suggest that molecular remodeling is initiated well before macro-level clinical changes become apparent. Previous studies have demonstrated that mRNA levels of *FOXJ1* and *RFX* family genes serve as reliable surrogate markers for ciliary density and function [26]. However, we acknowledge that further investigations utilizing single-cell RNA-seq (scRNA-seq) would be ideal to further dissect the cell-type-specific transcriptional dynamics, and high-speed video microscopy for ciliary beat frequency (CBF) and immunohistochemistry (IHC) for apical protein localization specifically in the post-dupilumab state are warranted to confirm the physiological kinetics of this recovery.

MUC5AC and MUC5B constitute the primary structural scaffolds of airway mucus, and their stoichiometric balance is a critical determinant of effective MCC. Consistent with reports in asthmatic patients [28,34,59], our study identified augmented MUC5AC expression and attenuated MUC5B levels in treatment-naïve ECRS nasal polyps (PreECRS). Notably, these expression profiles were not significantly modulated following dupilumab administration (PostECRS), suggesting that mucus properties in ECRS may be governed by regulatory axes independent of IL-4/13 signaling, such as human neutrophil elastase (HNE)-mediated mechanisms [60]. Within the functional modules of mucus production and rheology, the inclusion of POSTN, ALOX15, and SERPINE1 is particularly salient, as these factors synergistically drive mucus hypersecretion and viscosity. For instance, POSTN modulates the cellular protein expression of MUC5AC [33], while ALOX15 coordinates with IL-13 to regulate MUC5AC synthesis [30]. Furthermore, PAI-1 (encoded by SERPINE1) not only precipitates GCM but also exerts a potent inhibitory effect on fibrinolysis, thereby further compounding mucus viscosity [61,62]. While genes associated with “mucus production and secretion” were predominantly downregulated, and those related to “mucus rheology and quality” exhibited heterogeneous expression patterns, a distinctive trend was observed in “mucus degradation and remodeling”. Specifically, DNASE1L3—an endonuclease essential for the degradation of EETs, which significantly exacerbate mucus viscosity through DNA-mediated cross-linking —remained persistently downregulated post-treatment. It is important to consider that mucin expression programs may lag behind broader epithelial differentiation; thus, the lack of significant MUC5AC/MUC5B modulation at 4 weeks may reflect an ‘early-phase persistence’ of the mucus dysfunction. Further investigations with extended follow-up periods (e.g., 3–6 months) are warranted to determine whether sustained inflammatory control eventually facilitates the stoichiometric normalization of these structural mucins.

Additionally, the significant reduction in SPDEF expression was identified as a DEG in the PostECRS vs. PreECRS comparison. Since IL-13 ordinarily induces SPDEF via STAT6 signaling to drive GCM [31], our results suggest that targeted IL-13 inhibition reverses this pathological differentiation by attenuating STAT6 while concurrently restoring the ciliary master regulator FOXJ1. In conclusion, the interplay between aberrant mucus rheology and GCM represents a fundamental driver of mucociliary dysfunction in ECRS. While the pharmacological blockade of IL-4/13 signaling rehabilitates most of these pathological parameters, the deficit in mucus degradation and remodeling remains a therapeutic recalcitrance. Perhaps the most striking finding is the persistent downregulation of *DNASE1L3* in the PostECRS group, despite the clinical resolution of nasal polyps. The lack of recovery in this degradative pathway suggests that, at least within this early 4-week post-treatment period, the post-dupilumab mucosa remains in a state of “enzymatic recalcitrance”. Several limitations of this study must be acknowledged. First, the generalizability of our findings is constrained by the small sample size (*n* = 9), the single-center design, and the inclusion of only an East Asian population. Second, our results are based solely on transcriptomic data. Although we attempted proteomic analysis using the same samples, the expression levels of most study-associated proteins, including the ciliary master regulators (e.g., FOXJ1, RFX2/3), PCP-associated factors (e.g., WDPCP), and DNASE1L3, were below the detection threshold, precluding a robust comparative proteomic analysis. Furthermore, the study lacks functional validation, such as assessments of DNASE1L3 enzymatic activity, mucus rheology, ciliary beat frequency (CBF), or quantitative ciliary density via immunohistochemistry (e.g., FOXJ1 or acetylated alpha-Tubulin). Consequently, we cannot definitively distinguish whether the observed transcriptomic surge reflects an increase in the expression levels within individual cells or a broader shift in epithelial cell composition (i.e., an increase in the total number of MCCs). The ethical and technical challenges inherent in performing these measurements in vivo in human subjects represented a significant hurdle, limiting our ability to provide functional confirmation of our molecular findings. While these findings should therefore be interpreted with caution, this molecular deficit may explain why some patients, while clinically improved, still report highly tenacious mucus during viral infections or environmental triggers. Future studies utilizing in vitro air-liquid interface (ALI) culture models derived from these patients will be essential to bridge the gap between these transcriptomic ‘hardware’ signatures and objective functional recovery.

**Figure 5:**
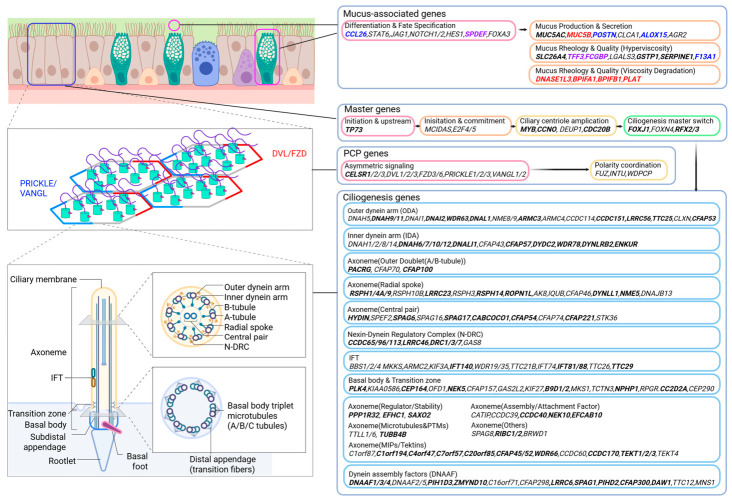
Detailed schematic linking gene expression modules to mucociliary epithelial architecture. This multi-scale schematic diagram integrates functional gene categorization with the hierarchical structure of a human mucociliary epithelium. Top-left: Illustration of a pseudostratified epithelium containing ciliated cells and mucus-producing goblet cells. A dashed-box inset shows a magnified view of the apical cell surface, highlighting Planar Cell Polarity (PCP) signaling complexes (PRICKLE/VANGL, DVL/FZD) involved in coordinating ciliary orientation. Bottom-left: Subcellular structural anatomy of a single motile cilium, detailed from the basal body and transition zone up through the central axoneme and tip. Magnified insets provide detailed schematic cross-sections of the 9+2 axonemal central pair arrangement (with labels for specific structural components) and the basal body triplet microtubule structure. Right and Bottom panels: expressed genes are functionally categorized into four hierarchical primary clusters: Mucus-associated genes: Divided into three modules: differentiation & fate specification, mucus production & secretion, and rheology & quality control (including handling hyperviscosity and viscosity degradation). Master genes: Stages of the ciliogenesis process, mapped sequentially from initiation & upstream signaling to commitment, centriole amplification, and the final ciliogenesis master switch. PCP genes: partitioned into modules for asymmetric signaling and polarity coordination. Ciliogenesis genes: an extensive, multi-category list organizing structural and regulatory components by their specific anatomical role (e.g., dynein arms, radial spokes, central pair, N-DRC, transition zone, IFT machinery). Lists of specific gene symbols are provided within each colored box. Genes shown in black or red bold indicate DEGs that are up- or down-regulated in the non-, pre-, and post-ECRS groups compared with controls. Purple bold indicates DEGs that show no significant differences from controls across the non-, pre-, and post-ECRS groups, but display significant downregulation after dupilumab administration (post- vs. pre-ECRS). Genes in blue bold denote those that are upregulated in the non-, pre-, and post-ECRS groups compared with controls but become downregulated following dupilumab administration. Abbreviations: PCP, Planar Cell Polarity; IFT, Intraflagellar Transport; N-DRC, Nexin-Dynein Regulatory Complex; ODA, Outer Dynein Arm; IDA, Inner Dynein Arm; DNAAF, Dynein Assembly Factors. Created in BioRender. In, T. (2026) https://BioRender.com/owlgr8u (accessed on 8 May 2026).

In summary, both non-ECRS and ECRS impair mucociliogenesis, with the severity being more pronounced in ECRS than in non-ECRS and controls. This enhanced impairment is attributable to stronger inflammatory damage in epithelial cells, accompanied by higher expression of epithelial cytokine–associated genes such as *TSLP* and *IL33*. Early modulation of mucociliogenesis following IL-4/13 blockade demonstrated that the recovery of the mucociliary respiratory epithelium is more rapidly promoted, involving a broad range of associated genes, including master regulators, planar cell polarity components, multiciliated-cell differentiation markers, and genes related to mucus properties (Figure 5).

On the other hand, most mucus-degradation factors remained consistently downregulated during the early phase of IL-4/13 blockade. These findings suggest that, although IL-4/13 inhibition effectively coordinates multiple facets of multiciliogenesis—including transcriptional regulation, mucus production, secretion, and rheological properties—it may exert only limited influence on the mechanisms governing mucociliary function. This limitation may stem from the persistence of high mucus viscosity, maintained by a chronically suppressed state, even when the mucosal surface appears clinically normalized following treatment. Our results support a model in which IL-4/13 blockade successfully rehabilitates the structural “hardware” of mucociliary function (cilia and planar cell polarity), while the enzymatic “software” responsible for mucus degradation (e.g., DNASE1L3) remains impaired. This “residual molecular scar” may account for the persistent mucus hyperviscosity observed in some ECRS patients despite clinical resolution of nasal polyps.

While these molecular and functional interpretations are based on transcriptomic profiling, further evaluation of actual DNase activity and mucus rheology is required to determine whether the sustained downregulation of *DNASE1L3* translates into clinically meaningful mucus hyperviscosity. Additionally, our study cohort has certain demographic limitations. Significant differences in height, weight, and sex were present among groups, which could act as confounding factors in cross-sectional comparisons. However, our principal conclusions rely on a longitudinal paired-sampling design (Pre-ECRS vs. Post-ECRS). By using each patient as their own internal control, we minimized inter-individual demographic variability and enhanced the resolution of IL-4/13 blockade-induced transcriptomic shifts. Nevertheless, we acknowledge that the small sample size and demographic imbalance are inherent to such a specialized clinical cohort, and future studies with larger and more homogeneous populations will be essential to validate the generalizability of our findings.

## 5. Conclusions

In conclusion, the present study elucidates the early transformative impact of dupilumab on the transcriptomic landscape of the sinus mucosa in patients with ECRS. Our findings demonstrate that the targeted inhibition of IL-13 orchestrates a rapid and early restoration of multiciliogenesis by upregulating the master regulatory hierarchy, specifically *FOXJ1*, *RFX2*, and *RFX3*. This early transcriptional recovery extends beyond mere structural assembly, encompassing the comprehensive activation of intraflagellar transport (IFT) complexes and axonemal motor proteins, thereby facilitating the holistic early reconstitution of ciliary transport and motility mechanisms.

Furthermore, we identified that transcriptional perturbations in PCP-associated genes—including the CPLANE module (*FUZ*, *INTU*, and *WDPCP*)—are significantly reversed as early as four weeks following dupilumab administration. Given the indispensable role of IL-33 as a clinical predictor of this early ciliary recovery, our results suggest that the early restoration of PCP architecture is a pivotal prerequisite for the re-establishment of coordinated MCC. Regarding mucus pathophysiology, while dupilumab effectively suppresses GCM through the early downregulation of *SPDEF*, it exerts a limited impact on the pathological stoichiometric balance of *MUC5AC* and *MUC5B* at this early stage. Specifically, the augmented *MUC5AC* and attenuated *MUC5B* expression levels observed in treatment-naïve ECRS remained largely unmodulated post-treatment, suggesting that mucus rheology in ECRS may be governed by regulatory axes independent of IL-4/13 signaling, such as HNE-related mechanisms. Notably, the persistent downregulation of *DNASE1L3* during the early phase of dupilumab administration underscores a continued deficit in mucus degradation and remodeling capacities, particularly concerning the clearance of EETs.

In summary, while IL-4/13 blockade successfully rehabilitates the structural and directional “hardware” of the epithelium, certain facets of mucus rheology and enzymatic degradation—the “software” of clearance—remain recalcitrant to therapy during the early phase. Future research should integrate high-resolution protein localization and functional kinetic analyses to achieve a more nuanced understanding of how these early molecular shifts translate into the clinical restoration of MCC. Ultimately, addressing residual impairments in mucus remodeling may pave the way for more comprehensive therapeutic strategies in the management of severe ECRS.

## Figures and Tables

**Table 1:** Patient demographics. Fractional exhaled nitricoxide: FeNO. Statistics were performed using one-way ANOVA and unpaired Student’s *t*-tests. N.S., not significant.

Mean ± SD	Ctrl	nonECRS	ECRS	*p* Value
Height	151.5 ± 4.0	165.8 ± 5.8	163.2 ± 8.4	*p* < 0.01
Weight	51.9 ± 6.2	63.2 ± 8.3	59.4 ± 6.2	*p* < 0.05
Sex (M/F)	0/6	5/3	5/4	*p* < 0.05
Age	61.5 ± 10.5	55.0 ± 10.5	52.9 ± 12.6	N.S.
Olfactory Distarbance (1:positive)	0.0 ± 0.0	0.3 ± 0.4	0.7 ± 0.7	N.S.
Blood Eosinophil	1.0 ± 0.5	1.7 ± 1.1	8.5 ± 6.3	*p* < 0.01
Total IgE (IU/mL)	231.5 ± 168.1	252.9 ± 300.1	302.1 ± 240.5	N.S.
Broncheal Asthma (1:positive)	0.0 ± 0.0	0.0 ± 0.0	0.8 ± 0.4	*p* < 0.0001
Lund-Mackay CT score		12.5 ± 6.0	18.0 ± 7.3	N.S.
Tissue Eosinophil (cell count/HPF)		21.1 ± 23.0	175.7 ± 46.1	*p* < 0.0001
JESREC Score		4.8 ± 3.3	14.9 ± 1.5	*p* < 0.0001
FeNO (ppb)		13.9 ± 6.1	50.9 ± 39.1	*p* < 0.05

**Table 2:** DEGs across study groups in (**A**) Master genes and (**B**) Mucus-associated genes. Gene Symbol: Standardized HUGO nomenclature for human genes. Category: Functional classification based on biological role in the mucociliary apparatus. “◯” represents upregulated DEGs and “▲” represents downregulated DEGs (*p_adjusted_* < 0.05; |log_2_FC| > 1).

**(A)**									
Genes	Main Category	Subcategory1	Subcategory2	PostECRS vs. PreECRS	nonECRS vs. Ctrl	PreECRS vs. Ctrl	PostECRS vs. Ctrl	PreECRS vs. nonECRS	PostECRS vs. nonECRS
*TP73*	Differentiation Regulators	Transcription factors	Initiation & Upstream				◯		
*MYB*	Differentiation Regulators	Multiciliogenesis	Ciliary centriole amplification				◯		
*CCNO*	Differentiation Regulators	Multiciliogenesis	Ciliary centriole amplification				◯		
*CDC20B*	Differentiation Regulators	Multiciliogenesis	Ciliary centriole amplification				◯		
*FOXJ1*	Differentiation Regulators	Transcription factors	Ciliogenesis Master Switch				◯		
*RFX2*	Differentiation Regulators	Transcription factors	Ciliogenesis Master Switch				◯		
*RFX3*	Differentiation Regulators	Transcription factors	Ciliogenesis Master Switch				◯		
**(B)**									
Genes	Main Category	Subcategory1	Subcategory2	PostECRS vs. PreECRS	nonECRS vs. Ctrl	PreECRS vs. Ctrl	PostECRS vs. Ctrl	PreECRS vs. nonECRS	PostECRS vs. nonECRS
*CCL26*	Differentiation & Fate Specification	Goblet Cell Metaplasia	Metaplasia-Driving Effector	▲		◯			
*SPDEF*	Differentiation & Fate Specification	Transcriptional Regulators	Goblet Cell Master	▲					
*MUC5AC*	Mucus Production & Secretion	Mucus Hypersecretion (Volume)	Mucin Synthesis			◯			
*MUC5B*	Mucus Production & Secretion	Mucus Hypersecretion (Volume)	Mucin Synthesis			▲	▲		
*POSTN*	Mucus Production & Secretion	Mucus Hypersecretion (Volume)	Mucin Upregulator	▲	◯	◯	◯		
*ALOX15*	Mucus Production & Secretion	Mucus Hypersecretion (Volume)	Secretion Inducers in combination with IL-13	▲	◯	◯	◯	◯	
*SLC26A4*	Mucus Rheology & Quality	Mucus Hyperviscosity (Quality)	Hydration & Homeostasis in combination with IL-4		◯		◯		
*TFF3*	Mucus Rheology & Quality	Mucus Hyperviscosity (Quality)	Mucin Stabilization	▲					
*FCGBP*	Mucus Rheology & Quality	Mucus Hyperviscosity (Quality)	Mucin Stabilization	▲					
*GSTP1*	Mucus Rheology & Quality	Mucus Hyperviscosity (Quality)	Mucin Stabilization				◯		
*SERPINE1*	Mucus Rheology & Quality	Mucus Hyperviscosity (Quality)	Fibrinolysis Inhibition			◯			
*F13A1*	Mucus Rheology & Quality	Mucus Hyperviscosity (Quality)	Fibrin Stabilization	▲	◯	◯	◯		
*DNASE1L3*	Mucus Rheology & Quality	Mucus viscosity degradation (Quality)	Chromatin-mediated Viscosity		▲	▲	▲		
*BPIFA1*	Mucus Rheology & Quality	Mucus viscosity degradation (Quality)	Hydration			▲	▲		
*BPIFB1*	Mucus Rheology & Quality	Mucus viscosity degradation (Quality)	Surface Lubrication & regulator of MUC5B			▲	▲		
*PLAT*	Mucus Rheology & Quality	Mucus viscosity degradation (Quality)	Fibrinolyisis Promotion/Plasminogen Activation			▲	▲		

## Data Availability

The raw data supporting the conclusions of this article will be made available by the authors on request.

## Supplementary Materials

The following supporting information can be downloaded at: https://www.mdpi.com/article/10.3390/cells15100911/s1, Table S1: Comprehensive gene lists; Table S2: Differential expression analysis, including (A) PCP genes, (B) Ciliogenesis genes, and (C) epithelial cytokines and receptors; Table S3: Linear mixed-effects model (LMM) results for treatment-induced longitudinal changes in IL-33 or TSLP; Table S4: Multivariate LMM analysis evaluating potential clinical confounders and baseline IL-33 interaction score.

## Abbreviations

15-HETE-PE: 15-hydroxyeicosatetraenoic acid-phosphatidylethanolamine; ALOX15: arachidonate 15-lipoxygenase; ANKRD6: ankyrin repeat domain 6; BRB-seq: Bulk RNA Barcoding and sequencing; CBF: ciliary beat frequency; CCNO: cyclin O; CDC20B: cell division cycle 20B; CELSR: Cadherin EGF LAG seven-pass G-type receptor; CPLANE: ciliogenesis and planar polarity effector; CPM: counts per million; CRSsNP: chronic rhinosinusitis without nasal polyps; CRSwNP: chronic rhinosinusitis with nasal polyps; DEGs: Differentially expressed genes; DEUP1: development of ependymal and urothelial cilia 1; DNASE1L3: deoxyribonuclease 1 like 3; DVL: dishevelled; E2F4/5: E2F transcription factor 4/5; ECRS: eosinophilic chronic rhinosinusitis; EETs: eosinophilic extracellular traps; ESS: endoscopic sinus surgery; FOXJ1: forkhead box J1; FOXN4: forkhead box N4; FUZ: fuzzy; Fz/Stan: Frizzled/Starry night; FZD: Frizzled; GCM: goblet cell metaplasia; HNE: human neutrophil elastase; IFT: intraflagellar transport; IHC: immunohistochemistry; IL-4Rα: interleukin-4 receptor alpha subunit; ILC2s: group 2 innate lymphoid cells; INTU: inturned; JESREC: Japanese Epidemiological Survey of Refractory Eosinophilic Chronic Rhinosinusitis; MCC: mucociliary clearance; MCCs: multiciliated cells; MCIDAS: multiciliate differentiation and DNA synthesis associated; MYB: MYB proto-oncogene; N-DRC: nexin-dynein regulatory complex; non-ECRS: non-eosinophilic chronic rhinosinusitis; PAI-1: plasminogen activator inhibitor-1; PCP: planar cell polarity; PLAT: plasminogen activator, tissue type; PPE: planar polarity effector; PRICKLE: prickle planar cell polarity protein; RFX: regulatory factor X; SERPINE1: serpin family E member 1; SPDEF: SAM pointed domain containing ETS transcription factor; STAT6: signal transducer and activator of transcription 6; t-PA: tissue-type plasminogen activator; TP73: tumor protein P73; TSLP: thymic stromal lymphopoietin; VANGL: Vang-like; WDPCP: WD repeat containing planar cell polarity effector.
